# Delivery of Community-based Antiretroviral Therapy to Maintain Viral Suppression and Retention in Care in South Africa

**DOI:** 10.1097/QAI.0000000000003176

**Published:** 2023-02-15

**Authors:** Melody Wang, Lauren R Violette, Jienchi Dorward, Hope Ngobese, Yukteshwar Sookrajh, Elliot Bulo, Justice Quame-Amaglo, Katherine K Thomas, Nigel Garrett, Paul K Drain

**Affiliations:** 1 Department of Global Health, University of Washington, Seattle, WA; 2 Department of Medicine, University of Washington, Seattle, WA; 3 Department of Epidemiology, University of Washington, Seattle, WA; 4 Centre for the AIDS Program of Research in South Africa (CAPRISA), University of KwaZulu-Natal, Durban, KwaZulu-Natal, South Africa; 5 Nuffield Department of Primary Care Health Sciences, University of Oxford, Oxford, Oxfordshire, United Kingdom; 6 eThekwini Municipality Health Unit, eThekwini Municipality, Durban KwaZulu-Natal, South Africa; 7 Department of Psychology, University of Washington, Seattle, WA; 8 Discipline of Public Health Medicine, School of Nursing and Public Health, University of KwaZulu-Natal, Durban, KwaZulu-Natal, South Africa

**Keywords:** HIV/AIDS, differentiated care, ART, viral load, point-of-care, viral suppression, retention in care

## Abstract

**Background:**

To determine whether the Centralized Chronic Medication Dispensing and Distribution (CCMDD) program in South Africa’s differentiated ART delivery model impacts clinical outcomes, we assessed viral load (VL) suppression and retention in care between patients participating in the program compared to the clinic-based standard of care.

**Methods:**

Clinically stable people living with HIV (PLHIV) eligible for differentiated care were referred to the national CCMDD program and followed for up to six months. In this secondary analysis of trial cohort data, we estimated the association between routine patient participation in the CCMDD program and their clinical outcomes of viral suppression (<200 copies/mL) and retention in care.

**Results:**

Among 390 PLHIV, 236 (61%) were assessed for CCMDD eligibility, 144 (37%) were eligible, and 116 (30%) participated in CCMDD. Participants obtained their ART in a timely manner at 93% (265/286) of CCMDD visits. VL suppression and retention in care was very similar among CCMDD-eligible patients who participated in the program compared to patients who did not participate in the program (aRR: 1.03; 95% CI 0.94–1.12). VL suppression alone (aRR: 1.02; 95% CI 0.97–1.08) and retention in care alone (aRR: 1.03; 95% CI 0.95–1.12) were also similar between CCMDD-eligible PLHIV who participated in the program and those who did not.

**Conclusion:**

The CCMDD program successfully facilitated differentiated care among clinically stable participants. PLHIV participating in the CCMDD program maintained a high proportion of viral suppression and retention in care, indicating that community-based ART delivery model did not negatively impact their HIV care outcomes.

## Introduction

The UNAIDS has established the 95-95-95 targets to end the HIV epidemic by 2030, with the goal of having 95% of all people living with HIV (PLHIV) who are accessing antiretroviral treatment (ART) maintaining VL suppression.^1^ Among the approximately 38 million PLHIV worldwide, roughly only half have achieved VL suppression, which is critical to reduce viral transmission and help avert the 650,000 deaths from AIDS-related illnesses reported in 2021.^2,3^ The World Health Organization recommends all PLHIV receive routine clinical assessments and VL testing to monitor therapy outcomes. As high volume clinics devote resources to diagnose HIV and initiate patients on ART, service delivery models that reduce the burden of unnecessary clinic follow-up visits are needed.^4^ Differentiated, community-based ART delivery models may reduce the burden on the healthcare system, while also giving clinically stable PLHIV greater access to ART.^5^

Almost one in five adults are living with HIV in South Africa, with an estimated 66% (5.1 million) PLHIV having suppressed VL in 2020.^6^ In 2014, the National Department of Health (NDoH) in South Africa established the Centralized Chronic Medication Dispensing and Distribution (CCMDD) program which is a public-private partnership that allows clinically-stable patients referred by healthcare workers in government clinics to collect ART refills at private pharmacies or community-based organizations.^7–9^ The CCMDD program supports patient ART adherence and retention by enabling convenient access to treatment at pick-up points, averting lengthy wait times at high volume HIV clinics, as well as diverting patients from overwhelmed clinic settings. CCMDD provides access to ART for over 1.7 million people and has become the largest differentiated care program in the world.^8,9^

However, there are mixed findings on whether decentralized community-based models of care results in similar patient loss to follow-up and adherence.^10–12^ Additionally, there is growing need to better understand how patients progress along the cascade of care to access ART through alternative delivery models in routine national programs. An evaluation of the delivery of community-based ART for maintaining viral suppression and retention in care in South Africa may establish the impact of CCMDD on clinical outcomes as well as better understand the patients who are able to enroll and access differentiated care. In this dataset from a trial cohort in Durban entering the national differentiated care program in South Africa under routine conditions, we sought to evaluate participation and identify gaps in the CCMDD program enrollment cascade, and ultimately determine the impact of CCMDD program participation on HIV care outcomes among clinically stable patients compared to standard treatment within health clinic facilities.

## Methods

### Study Design and Participants

Participant data in this analysis was collected from the STREAM (Simplifying HIV TREAtment and Monitoring) Study cohort, an open-label randomized controlled trial in Durban, South Africa from February 2017 to October 2018. Details of the research protocol have been published elsewhere.^13^ The study received ethical approval from the University of KwaZulu-Natal (BFC296/16) and University of Washington (STUDY00001466), and was registered with ClinicalTrials.gov (NCT03066128).

The study site was the CAPRISA eThekwini Clinical Research Site and the neighboring Prince Cyril Zulu Clinic, a large urban public clinic that provides HIV and primary care services for a diverse, mobile population of more than 10,000 patients. Eligible study participants were 18 years or older, living with HIV, and presented for their first routine HIV VL test six months after diagnosis. We excluded patients who were pregnant, had active tuberculosis, or required acute medical care by a physician. However, if participants became pregnant or were diagnosed with tuberculosis during follow-up, they were included in the analysis. Participants provided written consent and were enrolled in the STREAM parent study trial. After 6 months of follow-up in STREAM, study participants in both arms were then able to be assessed for enrollment into the CCMDD program as part of routine care for PLHIV in South Africa. Clinical outcomes across eligible participants who did and did not participate in the CCMDD program was measured and analyzed up to 12 months after STREAM enrollment.

### Study Procedures

The data used for the current study was collected as part of a randomized trial of quarterly point-of-care VL testing on retention in care and VL suppression (the STREAM parent study).^14^ At enrollment, we collected sociodemographic data, medical history, and potential risk factors for viral failure or loss to follow-up, such as method of transportation and distance to the clinic, HIV disclosure, ART adherence, and CD4 count. Mental health and substance abuse indicators were also collected, using the WHO Alcohol Use Disorder Identification Tool (AUDIT-C), WHO violence against women instrument (female participants only), and Patient Health Questionnaire-2 (PHQ-2) to screen for_depression. Participants received care according to South African HIV treatment guidelines, including testing for VL and creatinine at enrollment, and VL, creatinine, and CD4 cell count after six months. Any participant with a VL>1,000 copies per mL received enhanced adherence counselling and repeat VL testing after two months. If the repeat VL was >1,000 copies per mL, the participant was offered to switch to a standard second-line ART regimen. Participants in both comparison groups were reimbursed 100 South African Rand (ZAR) per clinic visit, per South African research guidelines.^13^

At least one year after initiating ART, stable participants in both arms of the trial qualified for the CCMDD program in South Africa, which allow clinically-stable participants to collect ART at decentralized pick-up points (PuPs).^9,15^ PuPs are contracted by the NDoH and include private pharmacies and community-based adherence clubs and organizations separate from clinic settings, as well as fast-track lanes at registered health facilities.^9,15^ Eligible patients may voluntarily register and select a PuP from which to collect medication every two months, and return to the clinic every six months for clinical review and prescription renewal.^15^ The NDoH distributes and dispenses medication through government tender across PuPs in eight provinces in South Africa. There is no cost for patients to participate in the CCMDD program.^15^

Participants were able to be assessed for CCMDD eligibility starting from 12 months post-ART initiation. Whether the participant was assessed for CCMDD eligibility as part of the national algorithm for differentiated care, and the outcome of this assessment, was captured in electronic CRFs by study staff. In accordance with South African guidelines, participants in both trial arms were eligible for referral into the program if they were non-pregnant, clinically stable on the same treatment regimen for at least 12 months, had CD4 count >200 cells per μL, no current TB, no uncontrolled diabetes or hypertension, and had two consecutive undetectable (<20 copies/mL) VL results.^16^ Once referred, participants collected ART every two months at a community pick-up point of their choice and were reviewed at the clinic by a professional nurse after six months. All blood testing occurred at the clinic. Participants not eligible for the CCMDD program or who chose not to participate continued with bi-monthly clinical visits. Any participant more than two weeks late for a scheduled clinic or community PuP visit received one phone call from the clinical team and resumed ART collection at the clinic, per South African guidelines.^13^ Laboratory and clinical records were used to assess the timing of participant referral into the community-based ART delivery program.

### Study Outcomes

The primary outcome for this analysis was a composite measure of retention in care with viral suppression, defined as <200 copies per mL, at 12 months. We used this composite outcome because both components are necessary to achieve positive treatment outcomes for adults living with HIV. Patient retention in care was defined as collecting ART at the study clinic or a community pick-up point between 44-56 weeks after enrollment. Participants not retained in care at 56 weeks were tracked by the study team up to 60 weeks for VL testing. All VL testing for the primary outcome was performed using a laboratory-based Cobas 6800/8800 machine (Roche, Basel, Switzerland). We also analyzed retention in care, viral suppression (each analyzed independently), and undetectable VL at study exit.

If a participant was ever formally assessed for CCMDD participation prior to the Month 12 study exit visit, they were considered “Assessed”. Of those who were considered “Assessed”, those formally deemed eligible prior to the Month 12 study exit visit were considered “Eligible”. Of those ever considered “Eligible”, participants formally referred to the CCMDD program prior to the Month 12 study exit visit were considered “Referred”. Among those referred, participants who have documentation that they picked up ART from a CCMDD pick-up point within the defined window prior to the Month 12 study exit visit are considered to have “Participated”.

### Statistical Analysis

The primary outcome was compared between two groups who were eligible for the CCMDD program: 1) Those who participated in the program and 2) Those who did not participate. Relative risks and 95% confidence intervals were estimated using relative risk regression via modified Poisson regression with robust standard errors. Our pre-specified analysis adjusted for study arm, continuous age, and sex in the final model based on prior literature. We conducted two sets of sensitivity analyses: (a) adding adjustment for distance from the clinic (<5km vs ≥5km), and high alcohol use, to ensure that these factors, which differed between groups by more than 7.2% (the equivalent of two participants in the smaller group), were not confounding our results, and (b) using a modified Poisson model with identity link to model risk differences (RDs) rather than RRs, in order to ensure that modeling a common outcome did not obscure the magnitude or importance of potential differences. Statistical significance was determined using a two-sided type one error rate of 0.05. Statistical analysis was performed using SAS 9.4.^17^

## Results

### Screening & CCMDD Program Eligibility

**Figure 1:**
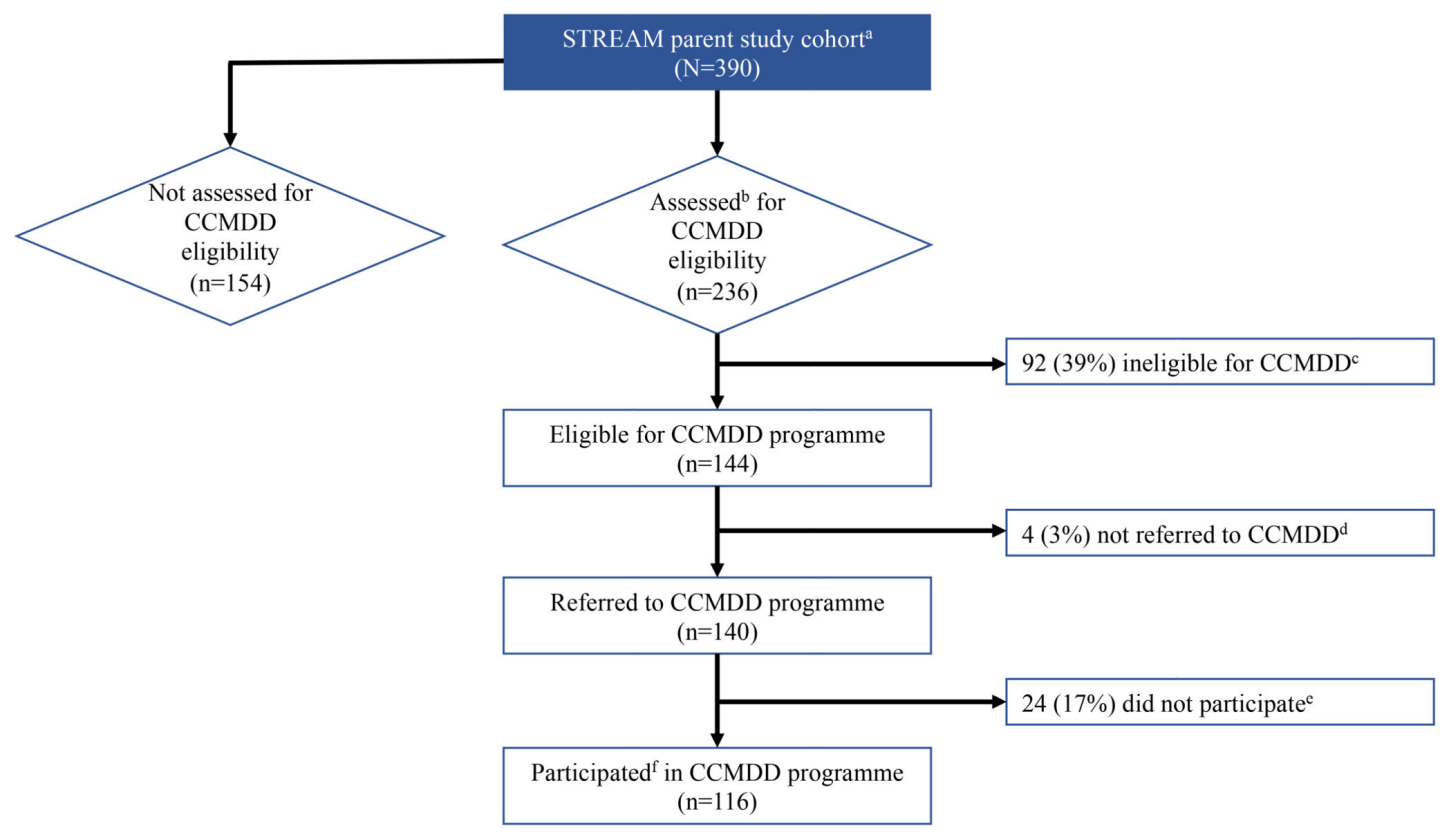
Study flow and cascade of care for participants from assessment to participation in the CCMDD program. Participation was determined by documented pick-up of ART from a CCMDD pick-up point within the correct window up to 56 weeks post-CCMDD enrollment and referral. a. STREAM trial study cohort on ART for minimum of 6 months. b. Participants assessed for CCMDD program on ART for minimum of 12 months. c. 39% (n=92) not eligible for CCMDD program. Reasons include: detectable VL (n=38), CD4 < 200 (n=20), not clinically stable (n=4), elevated/uncontrolled blood pressure (n=14), no valid identification (n=2), no record (n=5), pregnancy (n=3), weight loss (n=2), no consent (n=1), uncontrolled diabetes (n=2), 2^nd^ line medication (n=1) d. 3% (n=4) not referred for CCMDD program. Reasons include: CCMDD pick-up point full (n=2), no record (n=1), participant travels often (n=1) e. 17% (n=24) did not participate in CCMDD program. Reasons include: no record/did not attempt to get ART through CCMDD (n=22), missed appointment at pick-up point (n=2) f. Participation in CCMDD program followed for up to 56 weeks post CCMDD enrollment.

Between February 24^th^ 2017 and August 23^rd^ 2017, the study enrolled 390 PLHIV. Among study participants, 236 were assessed for CCMDD. Of these, 144 were deemed eligible, and 140 were formally referred to the CCMDD program (Figure 1). Of those who were referred to CCMDD, 83% (116/140) of participants participated in the CCMDD program. Among the STREAM study population, 63% (246/390 enrolled) were not assessed and/or eligible for the CCMDD program.

### Participant Characteristics

Across CCMDD-eligible and ineligible participants, there were no major differences in sociodemographic characteristics, travel time to clinic, HIV disclosure, and behaviors that could impact adherence to ART (Table 1). Among CCMDD-eligible participants, the median age of those participating in the CCMDD program was 30 years [IQR: 26–37] and 68% (n=79) were female, while the median age of those not participating in the CCMDD program was 32 years [IQR: 27-38] and 71% (n=20) were female (Table 1). Almost half (45%; n=52) of CCMDD program participants did not pass secondary school (Table 1). The majority of CCMDD participants also reported a monthly income greater than 1000 South African Rand (57%; n=66), had a stable partner (79%; n=91) and at least one child (79%; n=91).

### Gaps in the CCMDD program enrollment cascade

39% (154/390) of STREAM trial participants were not assessed for CCMDD program eligibility. Further investigation into baseline characteristics of patients not assessed showed they were similar to patients who were assessed for CCMDD eligibility, with a median age of 33 years (IQR: 27-38) and 34 years (IQR: 29-39), respectively. Public transportation was the main mode of transport to the clinic (88%), and the majority of unassessed patients had to travel at least 5 km to the health clinic (77%), though travel time was usually less than an hour (92%). Comparison of social-demographic characteristics, as well as mental health, drug/alcohol use, and ART adherence indicators, show this unassessed patient population was very similar to patients who were assessed, as well as patients deemed eligible for CCMDD (Table 1). The main difference observed was that unassessed participants had almost 2-fold higher prevalence of tuberculosis (21%) compared to assessed participants (12%) (Data not shown).

### CCMDD program yields very similar clinical outcomes compared to standard-of-care for clinically stable patients

At study exit after 12 months of clinical follow-up, 94% (109/116) of PLHIV who were eligible and participated in the CCMDD program had achieved both VL suppression and retention in care, compared to 93% (26/28) among those who were eligible but did not participate after adjusting for study arm, age, and sex (aRR: 1.03; 95% CI 0.94–1.12.) (Table 2). Results from the risk difference model were consistent: aRD: 0.03; 95% CI -0.06 to 0.11. These indicate very similar proportions consistent with at most a 6% lower probability of success in those participating in CCMDD. Results of the sensitivity analysis that also adjusted being ≥5km from clinic, and high alcohol use, yielded very similar results (aRR: 1.00; 95% CI 0.92-1.10). Among PLHIV who were not eligible, including those not assessed for CCMDD, 76% (188/246) were observed to achieve the composite outcome of VL suppression and retention in care at the Month 12 visit.

VL suppression alone (99% vs 96%, aRR: 1.02; 95% CI 0.97–1.08) or retention in care alone (95% vs 93%, aRR: 1.03; 95% CI 0.95–1.12) were also very similar between CCMDD-eligible PLHIV who participated and those who did not participate (Table 2). Among PLHIV who were not eligible for the CCMDD program, including those not assessed, 82.1% (n=202) were observed to achieve VL suppression and 85% (n=209) were retained in care by the Month 12 study exit visit. The proportion of participants with undetectable HIV VL (<20 copies/mL) was also similar between eligible groups who participated in CCMDD and those who did not, although this comparison rules out only at most a 19% lower probability of success (aRR: 0.96; 95% CI 0.81–1.14). Only 61% (n=150) of PLHIV who were not eligible, including those not assessed, for the CCMDD program had undetectable HIV VL at study exit visit. Sensitivity analyses for each of these outcomes produced similar results.

## Discussion

In this cohort of South African adults living with HIV, the majority of patients who participated in the CCMDD program picked up ART within the expected time window and remained eligible for the CCMDD program. Enrollment in differentiated care did not negatively impact clinical outcomes as levels of VL suppression and retention in care were equivalent between those who had received the standard-of-care compared to those who had participated in the CCMDD program. However, less than half of participants in the parent study were assessed for CCMDD program eligibility.

The observed effectiveness of CCMDD participation for maintaining the clinical outcomes of viral suppression and retention in care aligns with the expectation the CCMDD program would yield similar outcomes to the standard clinic-based ART provision in South Africa, and are consistent with findings reported in other studies across different global contexts.^18–22^ In these studies among stable adult patients, participants greatly preferred decentralized pick-up points and reduced clinic visits, leading to fewer patients lost to follow-up and better retention outcomes.^10,18,19,21^ However, community-based treatment is not always preferable to the clinic as there were also dissenting findings from a randomized control trial in South Africa that concluded that patients had better clinical outcomes in clinic-based adherence clubs compared to those in the community, though retention in both club models was poor.^11^ Our study adds to this growing body of evidence, and is one of the first to assess the impact of the CCMDD program on the clinical outcomes of viral suppression and retention in care.

Broad models of providing monitoring and treatment to millions of unique PLHIV are not likely to adequately support patients in the long-term, thus we must consider adapting HIV services to specific population needs and contexts. Reviews on different models of differentiated care which include facets of the CCMDD program such as community-based ART groups, decentralized and more infrequent ART pick-ups, and task-shifting, suggest that adapted ART delivery strategies are acceptable alternatives to traditional clinic-based care for PLHIV.^4,10,21,23^ However, access to these alternative service delivery models are often predicated on prior clinical stability from national guidelines, thus introducing a catch-22 where patients who demonstrate poor clinical outcomes of viral suppression are ineligible to participate in programs that may increase their access to medication. Although we cannot conclude whether CCMDD participation may improve clinical outcomes for those currently excluded, research is needed to further understand the mechanism by which PLHIV drop out of care and fail to suppress VL so that implementation of ART delivery strategies can be optimized.

Our findings also show that unassessed participants had almost 2-fold higher prevalence of tuberculosis compared to assessed participants. Current tuberculosis (TB) is an exclusion criteria for CCMDD, which may explain why these participants did not have assessment of CCMDD eligibility recorded in the study database. Studies have shown that among PLHIV with comorbidities, particularly TB, there were benefits to extending intervals between clinic visits and ART pick-ups from pharmacies, however, routine TB screening was still needed and risk assessment at treatment initiation to identify factors associated with poor clinical outcomes was recommended.^24,25^ Assessment and eligibility criteria may hamper access to the CCMDD program for patients who potentially have the most to gain from adaptive services. Additionally, there is also opportunity to use differentiated care not just for ART delivery, but for providing additional TB prophylaxis and screening that support both TB and HIV control measures.

Our study, and the CCMDD program in general, focused on non-pregnant, adult PLHIV who are stable on ART. This is a potential limitation of existing differentiated models of ART distribution. Key populations, such as adolescents, people who use injection drugs, and pregnant people, are particularly vulnerable to non-adherence and elevated VL.^26–29^ Lack of social support, stigma, and prior loss to follow-up events were identified as essential factors that negatively impacted retention in care in a cross-sectional study that recommended home and community-based care services be incorporated into HIV service delivery models.^30^ Further research is needed to determine whether widening access to such differentiated care services through the CCMDD program could provide these disenfranchised patients with multi-month refills and lower costs for patient travel and time through decentralized ART collection.

### Limitations

There were several limitations in this study, starting with the external validity of our study population. First, our study population consisted of participants from the STREAM trial, which reduces generalizability to other populations due to the types of people who are likely to join trials, and trial selection criteria. Second, retention in care in the STREAM parent study may be higher than the general population as all participants were reimbursed for study visits.^13^ In addition, there is potential for selection bias as our study did not randomize participants into enrollment for the CCMDD program. However, randomization of participants was logistically and ethically objectionable as CCMDD is a national program provided across South Africa and we studied the program as it was implemented.

Analysis of baseline socio-demographic characteristics as well as health behaviors among CCMDD-eligible participants who participated in the program and CCMDD-eligible participants who did not participate in the program did not show major differences. We have adjusted for observed differences in those who participated compared to those who did not, but unmeasured differences could remain. The trial intervention (point-of-care VL testing and task sifting to enrolled nurses) may have influenced assessment procedures for CCMDD and treatment outcomes, but we also adjusted for treatment arm in the regression model. Study reimbursements may also have impacted retention in care, however, identical incentives were provided for participants in comparison groups to minimize differences.

Our study reports outcomes six months following earliest possible CCMDD enrollment, thus there could be limitations as to how these findings translate to long-term retention in care. Some participants only participated in the CCMDD program for one month which may not be long enough to adequately capture changes to VL and retention in care. Further qualitative studies on patient preferences should also be conducted to determine feasibility and acceptability of the CCMDD program and provide data on barriers to participation. A relatively small sample size, particularly among eligible participants who did not participate in the CCMDD program, as well as the fact that participants are from a single clinical site serving an urban population of PLHIV in South Africa are also noted and may encourage further studies to determine outcomes across other contexts. These research gaps will be further explored in the STREAM HIV study currently conducted in both urban and rural clinics in KwaZulu-Natal, South Africa.^31^

### Implications & Conclusion

As universal access to ART becomes more established for millions of PLHIV worldwide, there is growing consensus that a blanket model of care is no longer an acceptable strategy for providing life-long HIV services. The CCMDD program in South Africa is an effective model for providing differentiated care for clinically stable patients, however, understanding and adapting ART delivery to unique patient contexts and needs is still needed to achieve undetectable VLs and long-term retention in care. There is a gap for a needs-based approach to servicing more vulnerable sub-populations unable to presently suppress VL, as opposed to a performance-based differentiated care model that caters exclusively to clinically stable patients.

In conclusion, we show that participation in the CCMDD program allowed stable adults living with HIV to suppress VL, be retained in care, and have undetectable VL levels that were similar to those who received standard-of-care treatment in a clinic-based setting. These findings support national implementation of CCMDD. Further research on the long-term effectiveness of the CCMDD program on VL suppression and retention in care is needed among representative sub-populations living with HIV, and not just among clinically stable patients.

## Figures and Tables

**Table 1:** Cohort by eligibility and participation in CCMDD program (N=390).

	Eligible for CCMDD program		Not Eligible for CCMDD program	
	Participated	Did not participate	Assessed	Not Assessed
	n=116	n=28	n=92	n=154
	n (%)	n (%)	n (%)	n (%)
**Socio-demographics**				
Median age - years [IQR]	30 [26, 37]	32 [27, 38]	34 [29, 39]	33 [27, 38]
Sex – Female	79 (68.1)	20 (71.4)	52 (56.5)	84 (54.6)
Highest level of education				
Did not pass secondary	52 (44.8)	11 (39.3)	49 (53.3)	81 (52.6)
Passed secondary	40 (34.5)	10 (35.7)	31 (33.7)	52 (33.8)
Tertiary or higher	24 (20.7)	7 (25.0)	12 (13.0)	21 (13.6)
Monthly income (ZAR)				
<1000	50 (43.1)	12 (42.9)	32 (34.8)	68 (44.2)
>=1000	66 (56.9)	15 (53.6)^c^	57 (62.0)^d^	80 (52.0)^e^
Has regular/stable partner	91 (78.5)	22 (78.6)	74 (80.4)	124 (80.5)
Children (1 or more)	91 (78.5)	22 (78.6)	76 (82.6)	127 (82.5)
Method of travel to clinic				
Walking	10 (8.6)	1 (3.6)	6 (6.5)	14 (9.1)
Public transportation	101 (87.1)	26 (92.9)	83 (90.2)	135 (87.7)
Private transportation	5 (4.3)	1 (3.6)	3 (3.3)	5 (3.3)
Travel >=5 kilometers to clinic	89 (76.7)	19 (67.9)	78 (84.8)	118 (76.6)
Travel < 60 minutes to clinic	108 (93.1)	28 (100.0)	81 (88.0)	142 (92.2)
**HIV Disclosure, mental health, and substance use**				
Disclosed HIV status to anyone	112 (96.6)	28 (100.0)	88 (95.7)	147 (95.5)
Positive Depression Screen ^a^	9 (7.8)	1 (3.6)	11 (12.0)	16 (10.4)
High Alcohol Use (Audit-C) ^b^	34 (29.3)	1 (3.6)	26 (28.3)	35 (22.7)
Current Tobacco smoking	15 (12.9)	2 (7.1)	19 (20.7)	28 (18.2)
Recreational drug use last 6 months	8 (6.9)	1 (3.6)	7 (7.6)	12 (7.8)
**ART adherence and CD4 testing**				
Self-reported 4-day ART adherence				
No missed doses	97 (83.6)	23 (82.1)	69 (75.0)	127 (82.5)
Missed any doses	19 (16.4)	5 (17.9)	23 (25.0)	27 (17.5)
Median CD4 count at enrollment [IQR]	536 [341, 719]	489 [367, 726]	348 [218, 587]	455 [314, 666]^f^
Study arm				
Standard of care	13 (11.2)	21 (75.0)	14 (15.2)	147 (95.5)
Intervention	103 (88.8)	7 (25.0)	78 (84.8)	7 (4.6)

a Depression is defined as a score greater than 1 on the PHQ-2.

b High alcohol use is defined as an Audit-C score of greater than 2 for women and greater than 3 for men.

c 1 participant that was CCMDD eligible but did not participate refused to disclose income.

d 3 participants that were not CCMDD eligible and were assessed refused to disclose income.

e 6 participants that were not CCMDD eligible and were not assessed refused to disclose income.

f 1 participant had missing CD4 count at enrollment.

**Table 2:** CCMDD participation prior to month 12 study visit and the association with HIV care outcomes.

	Participation in CCMDD prior to Month 12 visit		Adjusted RR^c^ (95% CI)	p-value	Adjusted RD^c^ (95% CI)	p-value
HIV care outcomes	Eligible but did not participate^a^ N=28 n (%)	Participated ^b^ N=116 n (%)				
HIV VL <200 copies/mL^e^ and retained in care at the study clinic at study exit ^d,e^ Yes	26 (92.9)	109 (94.0)	1.03 (0.94-1.12)	0.545	0.03 (-0.06, 0.11)	0.551
No	2 (7.1)	7 (6.0)	REF	-	REF	
HIV VL <200 copies/mL at study exit ^d^ Yes	27 (96.4)	115 (99.1)	1.02 (0.97-1.08)	0.404	0.02 (-0.03, 0.08)	0.397
No	1 (3.6)	1 (0.9)	REF	-	REF	
Retained in care at the study clinic at study exit ^e^ Yes	26 (92.9)	110 (94.8)	1.03 (0.95-1.12)	0.478	0.03 (-0.05, 0.11)	0.478
No	2 (7.1)	6 (5.2)	REF	-	REF	
Undetectable HIV VL at study exit ^d^ Yes	24 (85.7)	102 (87.9)	0.96 (0.81-1.14)	0.680	-0.03 (-0.19, 0.13)	0.700
No	4 (14.3)	14 (12.1)	REF	-	REF	

a Eligible for CCMDD but did not participate is defined as being appropriately assessed and eligible but has no documented receipt of ART from a CCMDD pick-up point on time at least once prior to the Month 12 study visit.

b Participation in CCMDD is defined as being appropriately assessed, eligible, referred, and has documentation of ART pick up from CCMDD pick-up point on time at least once prior to the Month 12 study visit.

c Relative risks and 95% confidence intervals were estimated using modified Poisson regression with robust standard errors. Risk differences and 95% confidence intervals were estimated using a risk difference (identity link) Poisson model with robust standard errors. Both types of models adjusted for study randomization arm, continuous age, and participant sex.

d HIV VL measured using Cobas 6800/8800 machine (Roche, Basel, Switzerland).

e Retained in care defined as collecting ART from the study clinic or CCMDD pick-up point between 44 and 56 weeks after enrollment.
